# Consonant Articulating Errors in Khmer-Speaking Preschool Children: A Picture-Naming Task Analysis

**DOI:** 10.1044/2026_JSLHR-25-00756

**Published:** 2026-10-02

**Authors:** Moeurn Linna, Tongquan Zhou

**Affiliations:** a School of Foreign Languages, Southeast University, Nanjing, China; b Institute for Language and Cognition, Southeast University, Nanjing, China

## Abstract

**Purpose:**

Consonant errors (mainly phonetic and phonological errors) as a phenomenon in speech development are frequently observed in preschool children's daily speech communication across languages. Since the Khmer language is a low-source language, consonant articulating error patterns of Khmer-speaking children have not been explored to date, leaving open the question whether the patterns are similar to those found in high-source languages such as English. To bridge the gap, this study investigated the on-spot speech of Khmer preschool children across three age groups (3;0 [years;months] to 5;11) using a picture-naming test with 203 pictures of varying complexity.

**Method:**

The naming data of 60 (34 girls and 26 boys) and 56 (34 girls and 22 boys) children, respectively, for two tests were recorded and transcribed for analysis, with errors categorized into (a) phonetic errors (substitution, deletion, addition, or distortion) and (b) phonological errors (regular and irregular).

**Results:**

The results showed that children aged 3;0–3;11 had the lowest speech sound accuracy among the three groups (3;0–5;11), with more frequent phonetic errors in substitution and deletion and more regular versus irregular phonological errors, including final consonant deletion, lateralization, initial consonant deletion, glottalization, and alveolarization substitution, but the errors did not reach statistically significant differences among the three age groups. The patterns are consistent with the developmental trends of speech sound error found in English and other high-source languages.

**Conclusion:**

These findings reveal the unique speech sound error patterns in Khmer preschool children, which may partly arise from linguistic universality during children's language development and partly be attributed to the typological features peculiar to the Khmer language.

**Supplemental Material:**

https://doi.org/10.23641/asha.33684361

Consonant articulating errors refer to the errors or difficulties arising from phonetic misarticulation and phonological difficulty in children's speech production (Brosseau-Lapré & Schumaker, 2020; Namasivayam et al., 2020). These difficulties pertain to the most common types of communication disorders in early childhood (Preston et al., 2014) and appear to be associated with reduced speech intelligibility and negative social, personal, academic, and educational factors (McCormack et al., 2009). Obviously, a good understanding of the speech sound errors plays an important role in promoting accurate speech production and supporting broader language acquisition.

Despite the growing literature on speech sound errors in children of different language backgrounds, there have been no similar studies on Khmer-speaking children, hindering the systematic recognition of Khmer native speakers' speech development. Prior research has consistently shown that preschool children acquire vowels earlier and more accurately than consonants (Bernthal & Bankson, 1998; McLeod & Crowe, 2018), although consonants are at a higher risk of production difficulties, particularly those with complex articulatory demands (Bankson et al., 2017). Against this background, it is of great significance to conduct an in-depth investigation of consonant errors in Khmer-speaking preschool children, aiming to analyze phonetic and phonological errors (i.e., speech sound errors in general).

## Consonant Articulating Errors in Language Development

Consonant articulating errors are often observed in children with functional speech sound disorders during early language development (Brosseau-Lapré & Roepke, 2019; Preston & Edwards, 2010). Children tend to exhibit the majority of difficulties in three core domains, such as perception, motor production, and phonological representation of speech sounds and segments (American Speech-Language-Hearing Association [ASHA], 2014). These difficulties become the challenges for children to accurately produce and contrast speech sounds because they fail to establish the motor and cognitive–linguistic processing chain involved in speech production (Berti et al., 2022; Brumbaugh & Smit, 2013; Priester et al., 2009; Wertzner et al., 2017). Moreover, consonant errors occurred more frequently in children than other communication errors (e.g., speech impairments, speech disorders, and speech mistakes) associated with social and academic difficulties (Campbell et al., 2003; Fox & Dodd, 2001; Mullen & Schooling, 2010; Shriberg et al., 1999). For instance, Shriberg et al. (1999) found that 3.8% of 6-year-old children struggled with their consonant errors, whereas Campbell et al. (2003) showed that the errors affected 15.6% of 3-year-olds and 6-year-olds. These investigations demonstrate that children's speech sound errors commonly occurred in early childhood.

Consonant errors occur when a child's speech is obviously different from that of surrounding peers. The errors fall into four main classifications (surface structurers) in light of Dodd and McCormack's (1995) description for English-speaking children: (a) phonetic errors (producing correct sounds due to incorrect motor program), (b) delayed phonological skills (when a child uses speech patterns much later than their corresponding normal children), (c) consistent phonological errors (when a child makes one speech sound error repeatedly), and (d) inconsistent phonological errors (when a child makes a speech sound error irregularly each time, namely, without a fixed pattern; also see Berti et al., 2022; Dodd et al., 1997; Lewis et al., 2006; Liu & Chien, 2020). These classifications have been proven to be valid in languages other than English (Kirk & Vigeland, 2015; Lam & To, 2017). Other classification systems have also been developed, such as those proposed in ASHA (2014), Brosseau-Lapré and Roepke (2019), Lewis et al. (2006), Shriberg (2010), and Waring and Knight (2013). As a shared point, these classifications were established by referring predominantly to European languages. Moreover, from different perspectives, consonant articulation errors were subtly categorized into more subtypes, such as those regarding speech delay–genetic, speech delay–otitis media with effusion, speech delay–apraxia, speech delay–dysart, and speech delay–developmental psychosocial involvement (Lewis et al., 2006; Nojavan-Pirehyousefan et al., 2022; Shriberg et al., 1997, 2005). However, these categories generally did not cover regular speech sound errors in verbal communication, for instance, failing to distinguish between phonetic and phonological errors particularly.

Consonant articulating errors appear when children learn to articulate speech sounds. Most of these errors have no known causes (ASHA, 2014). However, studies have indicated that at least 70% of children with speech sound errors are directed to pediatric speech pathologists for treatment (Nathan et al., 2004). While the exact cause of speech sound errors in children remains unknown, numerous studies (e.g., Alderete et al., 2021; Kim et al., 2025; Nijland, 2009; Rvachew & Grawburg, 2006; Salvago et al., 2019) have attempted to understand how individual consonant articulating errors affect the manner in which sounds are produced. Such errors may reduce speech intelligibility (Anthony et al., 2011; Gierut, 1990) and be linked to difficulties in social development, achieving less in school, and even struggling to support themselves later in life (Lewis & Freebairn, 1992). All these points indicate that speech sound errors have profound impacts on children's lives.

## Speech Sound Patterns of the Khmer Language

Cambodian, also known as Khmer, is Cambodia's official language, with around 16–20 million speakers (Donley, 2020; Eberhard et al., 2015). Khmer belongs to the Khmer-Mon language family, a subbrand of the Austro-Asiatic language, exhibiting more complex speech sound patterns (including both phonetics and phonology) than many other languages within the same family. Unlike most Austro-Asiatic tonal languages, Khmer is stress-based (Tikkanen, 2010).

The Khmer speech sound system is composed of 17 consonants (see Appendix A) and 18 vowels (Filippi & Hiep, 2016). Consonants are classified by their place and manner of articulation, while vowels are classified based on tongue height, tongue position, and lip rounding (Filippi & Hiep, 2016). Khmer consonants are further categorized into two distinct series (i.e., ɑː-series and ɔː-series) based on how consonants collocate with their surrounding vowels (e.g., Donley, 2020; Filippi & Hiep, 2016; Huffman, 1967). The two series are composed of stops, nasals, and fricatives and articulated across bilabial, alveolar, palatal, velar, and glottal places of articulation. This contrast plays a crucial role in the Khmer phonological system. The vowel system also adopts a dichotomy of speech sounds, namely, dependent and independent vowels, together constituting a rich inventory of monophthongs and diphthongs in Khmer language (Soky et al., 2016).

Phonetically, Khmer is characterized by a relatively complex system of consonant clusters. According to Donley (2020), the language is composed of 87 possible consonant clusters, 85 with two consonants and two with three consonants (e.g., Huffman, 1987; Kirby, 2014). These clusters occur only in word-initial syllables and are generally absent in the word-final position, except in loanwords (Kirby, 2014). Khmer syllables are generally constructed by adopting five structure patterns: consonant–vowel, CV (e.g., [baː]), CCV (e.g., [trɛj]), CVC (e.g., [maʔ]), CCVC (e.g., [prɛeh]), and CCCVC (e.g., sthɜt; Choosri, 2009; Huffman, 1987). Notably, even the maximal syllable structure (i.e., CCCVC) is simpler than many complex English forms (i.e., CCCVCCC), as illustrated in [streŋθs] (Donley, 2020).

Several Khmer consonants are phonetically different from those used in English. For example, the voiced palatal stop /c/ in Khmer does not correspond to the English affricative /tʃ/, and the voiceless labiodental fricative /f/ is found only in borrowed terms. In contrast, English includes additional fricatives, such as /v, θ, ð, z, ʃ, ʒ/, which are not present in Khmer (Dyson & Amayreh, 2000). These phonetic differences may influence how speech sound patterns emerge during development, as shown in comparative studies (Dyson & Amayreh, 2000; Nojavan-Pirehyousefan et al., 2022).

In contrast to high-source languages such as English with abundant and in-depth exploration of speech sound errors in early language acquisition, Khmer, as a low-source language, is purely a barren landscape to be cultivated in this regard. While Khmer has been described in terms of its phonological inventory and adults' speech patterns (e.g., Choosri, 2009; Haiman, 2011; Kirby, 2014; Wayland & Jongman, 2003), no study has been conducted on preschool children's consonant articulating errors in Khmer, leaving the issue unexplored as to what comprises the picture of speech production patterns in Khmer-speaking preschool children.

To bridge this gap, this study undertook the first endeavor to examine the consonants' phonetic and phonological error patterns by Khmer preschool children using picture-naming tasks. Therefore, this study directly addresses the lack of systematic description of speech sound errors in Khmer to provide fundamental data to enrich linguistic sources in the existing literature (McLeod & Crowe, 2018; Nojavan-Pirehyousefan et al., 2022).

## The Present Study

Against this background, the present study was conducted using picture-naming tasks to investigate the consonant articulating errors of Khmer-speaking preschool children and to analyze the potential factors that motivate their speech difficulties. By the way, the collected data regarding Khmer phonetic and phonological errors were used to make a comparison between Khmer and other languages from the perspective of speech configuration. Specifically, this study addressed the following two questions:

1. How do the Khmer-speaking preschool children perform in articulating Khmer consonant errors?
2. What are the patterns of consonant articulating errors by the Khmer-speaking preschool children across age groups?

## Method

### Ethical Statement

Ethical approval was received from the Ethics Committee of the School of Foreign Languages and Literature at Southeast University (China), with institutional review board Approval No. 00001868, prior to the present study. The data collection from schools was approved by the Cambodia Ministry of Education, Youth, and Sport, with Approval No. 211. The consent written forms were obtained from the participant's family members, teachers, or caregivers, respectively.

### Participants

Sixty Khmer preschool children (34 girls and 26 boys) were recruited from three state kindergarten schools in the downtowns or areas surrounding the provinces of Cambodia, each with informed consent from their guardians, such as parents, teachers, or caretakers. Their average age was 4;0, ranging from 3;0 to 5;11 (*M*_age_ = 4.04, *SD* = 0.81). According to their parents, all the children spoke only Khmer and had no speech disorders, language delays, developmental challenges, or oral–facial anomalies. None of them had received speech therapy before this participation. All children were classified into three age groups: 20 for each (i.e., 3;0–3;11, 4;0–4;11, and 5;0–5;11). All participants were selected randomly depending on the inclusion criteria. Additionally, they all passed an oral phonetic test and hearing screening test of ≤ 20 dB HL at 100, 200, and 4000 Hz prior to the experiment (also see Berti et al., 2022; Govaerts et al., 2006; Preston & Edwards, 2010).

The inclusion criteria included four aspects. Specifically, all the recruited children:

1. were of Cambodian ethnic origin and had Cambodian parents;
2. were aged 3;0–5;11 and had no reported mental, physical, syndromic, or hearing problems;
3. attended education at a local kindergarten school in the downtown areas of Cambodia and started preschool from 3;0 to 3;5, having at least 6 months of consistent Khmer exposure in the preschool; and
4. exhibited behaviors such as playing alone or rarely speaking with anyone, stuttering, crying, and speaking too much, among others (as reported by teachers, parents, and testers).

Based on these inclusion criteria, 60 participants were enrolled in this study. However, four children uttered minimal speech sounds (i.e., they named too few images to provide a sufficient speech sample), and 56 (34 girls and 22 boys) completed the naming tests as required; hence, their data were adopted for the final analysis.

All participants were classified as typical children with unknown speech sound errors (phonetic and/or phonological errors for unknown reasons) in terms of an internationally agreed testing protocol. First, the children were scored at or below the 10th percentile on the Goldman-Fristoe Test of Articulation Sounds in Words, indicating moderate to severe impairment (ASHA, 2014; Fudala, 2000; Goldman & Fristoe, 1969). Second, each child produced at least three phonological processing errors in light of the Khan–Lewis Phonological Analysis (Khan & Lewis, 1986). Third, receptive vocabulary was age appropriate, as indicated by the Receptive One-Word Picture Vocabulary Test (Brownell, 2001; Martin & Brownell, 2011). Fourth, conversational samples showed percentage of consonants correct–revised scores below 90% (Shriberg et al., 1997).

All the recruited 56 children had an average consonant accuracy of less than 90% (see Figure 5). The general evaluation in ASHA (2014) was adopted to grade participants' speech sound errors, including the evaluation of accurate productions, speech sound errors, and error patterns.

### Instrument and Procedure

This study adopted a picture-naming task, as previously adopted by Dyson and Amayreh (2000), to investigate how preschool children uttered speech sounds in Khmer (their native language) to name pictures placed in front of them. Testing materials have demonstrated their validity in a growing body of research on speech sound errors in preschool children (e.g., Biran et al., 2023; Liu & Chien, 2020). The entire test consisted of 203 color pictures that were designed to cover all the consonants and vowels in Khmer, ranging from simple to complex sound patterns (Appendix B and Supplemental Materials S1 and S2). Furthermore, the test words were controlled on the basis of the phonetic and phonological inventory of Khmer, each comprising one to four syllables and two to 10 phonemes, so that the named words are those frequently used in children's daily speech.

During the test procedure, children were invited to sit individually in a quiet room and were asked to name each picture shown by a tester^1^ (experimenter) with the direction, “Please tell me what you see in the picture, or do you know the name of this picture?” After each child uttered a name for the target picture, he or she was given positive feedback, regardless of whether the naming was correct or wrong. There was no time limit for naming each picture. All naming tests were recorded for post hoc reexamination of the uttered speech sounds. During the procedure, if a children responded with “I don't know” or kept silent for a several seconds after initial instruction, the tester offered a further prompt, “Listen to me how I read the target word (e.g., [ɲam] ‘eat’).” If the child still did not respond, the tester requested the child to repeat or imitate the word two or three times. This step-by-step procedure was designed to decrease the number of words that had not been uttered. In this situation, the tester counted speech sound errors when the child could not repeat words.

After all tests were completed, the data were transcribed into words according to the International Phonetic Alphabet (IPA). The transcriptions were checked by two Khmer phonetic and phonological experts from the Department of Linguistics, with the interrating an accuracy and reliability 98.99%. Specifically, for the transcription consistence, the two transcribers listened to the speech data for two or more times, particularly upon encountering noisy speech files recorded.

On average, each participant produced 118 single words and 85 multiword words, which involve 532 consonant and 299 vowel tokens, covering all consonant and vowel phonemes of the Khmer phonetic inventory in three positions: word-initial, word-medial, and word-final. All Khmer consonants were collected from the naming tasks wherein at least one token occurred in each of three positions, including /b/ (17), /c, cʰ, cᵊ/ (31), /d/ (24), /h/ (16), /j/ (14), /k, kᵊ, kʰ/ (86), /l, lᵊ/ (31), /m, mᵊ/ (30), /ɲ/ (5), /n/ (29), /ŋ/ (34), /p, pʰ/ (37), /r/ (43), /s, sᵊ/ (43), /t/ (58), /v/ (6), /ʔ/ (28) (*M* = 31.29, *SD* = 19.62, *n* = 532). All elicited speeches were recorded using an Olympus Voice-Trek VN-702PC, a Sony IC Recorder ICD-UX512F, and an mrobo digital voice recorder, RGH High FrideLity, and were phonetically transcribed by the first author using the Bluetooth Headphone A2DP/AVRP/HFP/HSP in a quiet room.

### Coding Data and Analysis

All of the naming data were coded in the following stages: In the first stage, each participant's data were transcribed into an Excel file using the IPA for analysis of the speech sound error patterns and error types. Afterward, a measurement of the consonants' production accuracy was conducted by having the participants' correctly produced consonants divided by all the consonants they produced in the test.

In the second stage, all consonant articulating errors were classified into two categories, phonetic and phonological errors, following the general principle outlined by ASHA (2014) and Dodd and McCormack (1995) and other language studies by Dyson and Amayreh (2000), Liu and Chien (2020), and Nojavan-Pirehyousefan et al. (2022). For the phonetic error type, consonant errors were categorized into the following subgroups: substitution (e.g., [kʰlɘɨ] > [hvʰɘɨ]), deletion (e.g., [kʰmʊm] > [mʊm]), addition (e.g., [cʊndaɜ] > [cɒmkᵊdaɜ]), and distortion (e.g., [s] > [skʰ]). For phonological error type, speech errors were categorized into two subgroups: regular and irregular phonological errors. Specifically, regular phonological errors were frequently produced by children across all age groups (e.g., weak syllable omission [ʔɑndaːt] > [daːt]). In contrast, children of all age groups rarely produced irregular phonological errors (e.g., backing of stopping [t] > [k]).

In the third stage, the number and proportion of participants regarding each error pattern were calculated. Similarly, the number of all speech errors was divided by the number of consonants uttered by the children to obtain the frequency of each error pattern before all data were pooled for statistical analysis using SPSS and the Data Analysis Tool in Excel Version 2311 (Build 17029.20108 Click-to-Run). Additionally, descriptive statistics, including the mean and standard deviation, were used to demonstrate consonant articulating errors in Khmer-speaking preschool children. One-way analysis of variance (ANOVA), repeated-measures ANOVA, and chi-square were adopted to compare the general error performances in producing Khmer consonants error and their corresponding error patterns among the three age groups. These methods were selected since they handle variations from normality and homogeneity of variance well, with the significance threshold set at *p* < .05. When the general test was significant, the Bonferroni-pairwise comparison was conducted to identify which age groups differed from each other.

## Results

As shown in Figure 1, Khmer preschool children produced more phonetic errors (*M* = 82.99, *SD* = 105.77) than phonological errors (*M* = 90.57, *SD* = 130.07),^2^ with each error type accounting for approximately 57.58% and 42.42% of the total consonant articulating errors, respectively (see Figure 1). However, the main effect of error type was statistically insignificant, *F*(1, 199) = 0.20, *p* = .65, η^2^ = .01. What follows describes the phonetic and phonological errors using one-way ANOVA, repeated-measures ANOVA, and chi-square, each with a presentation of general performance and corresponding error patterns.

**Figure 1.**
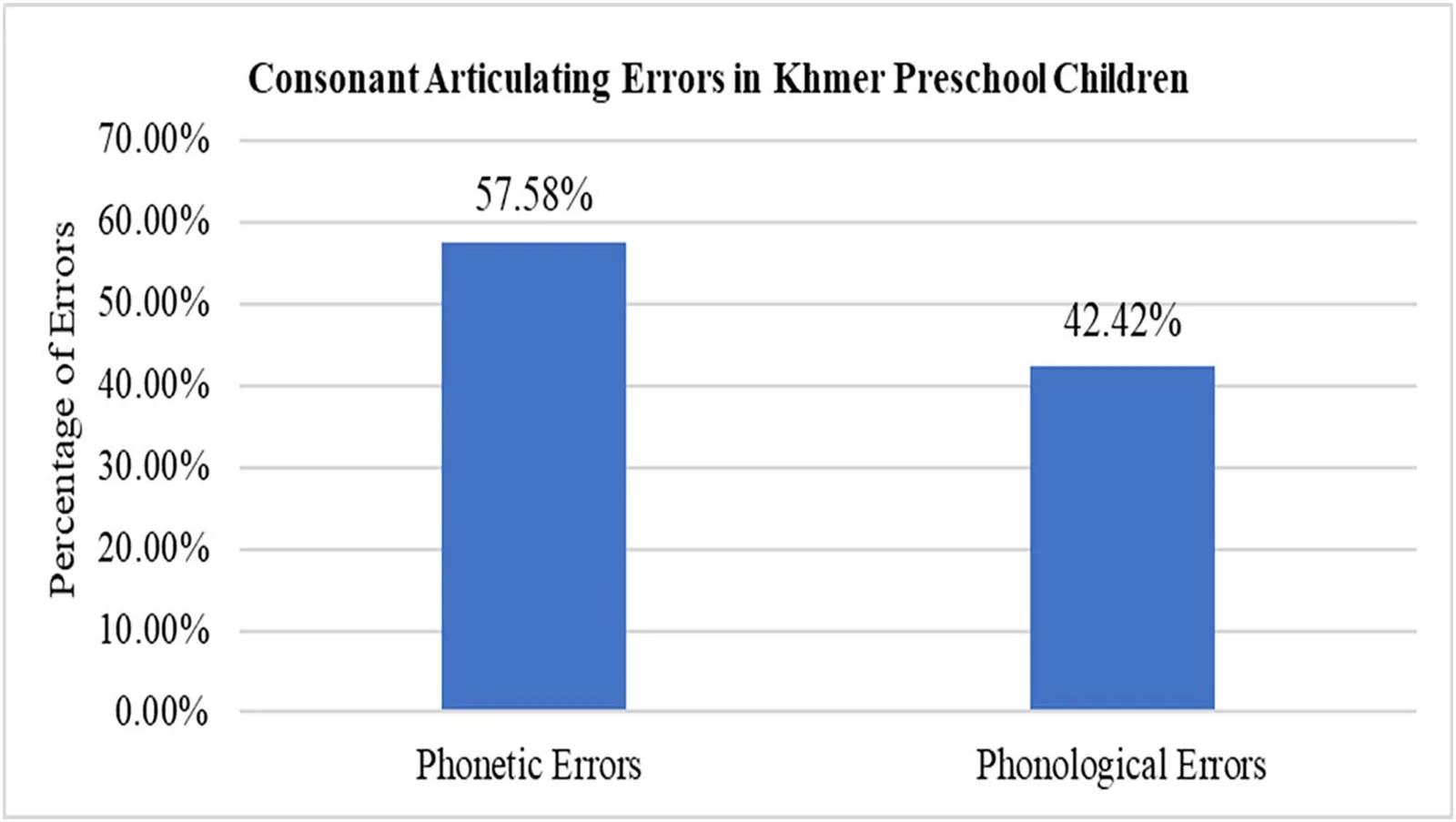
Percentage distribution of phonetic and phonological errors for Khmer preschool children.

### Consonant Phonetic Errors Across the Three Age Groups

#### General Performance in Consonant Phonetic Errors

Our data showed that Khmer preschool children tended to make four types of phonetic errors, including substitution, deletion, addition, and distortion. Since addition and distortion errors basically accounted for less than 1% of the total errors, the following report was just centered on substitution and deletion errors, with their average proportion displayed in Table 1.

**Table 1.** Phonetic consonant errors in the Khmer preschool children.

Error types	3;0–3;11 (*M* ± *SD*, *n* = 20)	4;0–4;11 (*M* ± *SD*, *n* = 20)	5;0–5;11 (*M* ± *SD*, *n* = 20)	*F* (*df*)	*p* value
Substitution	122.2 ± 127.20	68.55 ± 52.13	62.45 ± 111.22	2, 57 = 2.07	.13
Deletion	121.75 ± 130.68	63.1 ± 54.75	58.45 ± 111.78	2, 57 = 2.31	.10

*Note.* The percentages of each error type were calculated by dividing the number of consonant errors by the total number of consonants acquired between ages 3;0 and 5;11 (years;months).

The descriptive statistical analysis revealed that younger children aged 3;0–3;11 had the highest errors in substitution (*M* = 122.20, *SD* = 127.20) and deletion (*M* = 121.75, *SD* = 130.68), with each type constituting approximately 48%–50% of the total errors (see Figure 2). However, the corresponding error rates decreased in older children: Those aged 4;0–4;11 presented the lowest substitution (*M* = 68.55, *SD* = 52.13) and deletion rates (*M* = 63.10, *SD* = 54.75), while the age of the 5;0–5;11 group had the fewest errors overall (substitution: *M* = 62.45, *SD* = 111.22; deletion: *M* = 58.45, *SD* = 111.78).

One-way ANOVA was used to compare substitution and deletion errors across the three age groups. The results revealed no significant error type difference between substitution, *F*(2, 57) = 2.07, *p* = .13, η^2^ = .06, and deletion, *F*(2, 57) = 2.31, *p* = .10, η^2^ = .07, across all age groups. Despite the absent of significance, there appeared a trend that younger children (aged 3;0–3;11) produced more phonetic errors than older children (aged 4;0–5;11).

**Figure 2.**
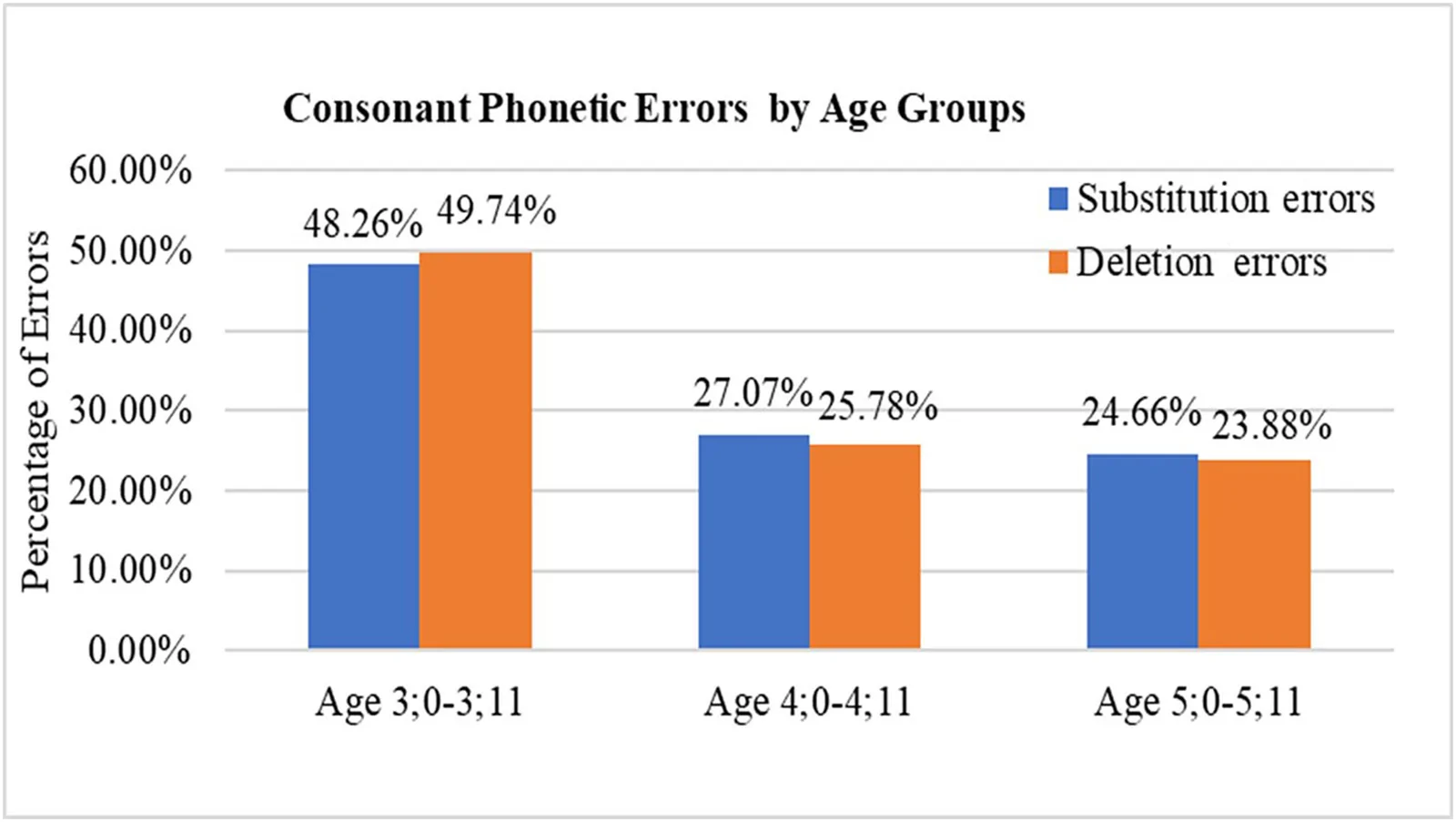
Percentage of substitution and deletion errors by three age groups of Khmer-speaking children.

In brief, the above findings showed a general developmental pattern in phonetic accuracy, with younger children showing more errors than older children. However, the absence of statistically significant group differences revealed that individual variation remained high during this developmental period.

### Patterns of Consonant Phonetic Errors

Table 2 presents the percentages of consonant phonetic errors, which reflect the hierarchy of difficulty in Khmer consonants' production. The general pattern of consonant phonetic error by the Khmer preschool children can be revealed from two dimensions, the error type and place of articulation.

**Table 2.** Phonetic consonant error patterns across age groups in Khmer-speaking preschool children.

Error types	3;0–3;11	4;0–4;11	5;0–5;11
Substitution	b (3.86), d (5.84), c (6.19), r (15.23), s (17.34), t (6.86)	b (4.67), d (8.13), c (9.34), r (15.59), s (17.47), t (7.83), k (27.22)	b (5.71), d (4.24), c (10.36), r (9.05), s (16.96), t (7.26), k (13.30)
Deletion	b (2.25), d (3.05), c (6.80), n (5.79), r (26.26), t (11.03)	b (1.18), d (1.18), c (3.15), n (4.65), r (28.08), t (7.80), k (17.42)	b (1.82), d (2.27), c (3.37), n (6.38), r (26.16), t (9.57), k (12.30)

*Note.* Value in the parentheses represent the percentages of total errors for each consonant in the specified age group. Only consonants with higher error frequencies are listed. Age groups are categorized as follows: 3;0–3;11, 4;0–4;11, and 5;0–5;11 (years;months).

In terms of error type, the pattern of phonetic errors across all the children can be described as substitution > deletion > addition & distortion (> meaning “more errors than”). This suggests that children made more substitution error (54.36%; *M* = 296.48, *SD* = 264.35) than deletion errors (45.64%; *M* = 248.88, *SD* = 271.87), as mentioned above. However, the chi-square test did not reveal the significant difference within these phonetic errors (*p* = .23), suggesting consistency in their occurrence within this age group. More specifically, children aged 3;0–3;11 exhibited the highest frequencies of both substitution (*M* = 98.82, *SD* = 91.78) and deletion (*M* = 82.96, *SD* = 94.77) errors, but the older age groups (aged 4;0–5;11) displayed a significant reduction in these errors (substitution: *M* = 72.74, *SD* = 69.28; deletion: *M* = 69.56, *SD* = 81.06; see Table 2 for the distribution of phonetic error patterns across age groups; also see Supplemental Materials S1 and S2).

Since the data set was composed of 2 (substitutions and deletions) × 3 (aged: 3;0–3;11, 4;0–4;11, and 5;0–5;11), a repeated-measures ANOVA was performed to analyze the consonant phonetic errors across the three age groups. The results indicated significant group differences in the consonant phonetic error patterns (i.e., substitution > deletion). Specifically, substitution errors showed a main effect, *F*(1.33, 21.39) = 13.40, *p* < .00, η^2^ = .24, with significant difference among aged 3;0–5;11 (all *p*s < .00). However, no significant differences were found among aged 4;0–5;11 (all *p*s = .91). Similarly, deletion errors also showed a main effect, *F*(1.26, 20.14) = 5.14, *p* < .02, η^2^ = .24, although there were no significant across age groups (all *p*s = .18). These findings illustrate an age-related progression in consonant acquisition, suggesting that articulation skills advance progressively with age, with younger children facing greater phonetic instability.

In terms of place of articulation, substitution errors occurred more frequently in alveolar (12.61%), palatal (8.63%), and velar (20.26%) consonants, whereas deletion errors occurred more frequently in alveolar consonants (13.97%) roughly comprising the pattern: velar consonant > alveolar consonant > palatal (> meaning “more errors than”). This suggests that consonants requiring fine articulation control or complex tongue positioning are prone to deletion during children's early utterance. Additionally, the persistence of high rates of alveolar trill error (20.05%) among the observed phonetic errors indicates its status as a pronounced segment in Khmer phonology. This finding is consistent with cross-linguistic evidence of the delayed acquisition of rhotics (Alfwaress et al., 2015; Solé, 2002; Yavaş & Kehoe, 2024; Yavas & Lamprecht, 1988).

In summary, these phonetic error patterns underscore a clear age-related reduction, showcasing consonant-specific vulnerabilities that appear to be tied to both articulatory difficulties and language-specific phonotactic constraints. The general patterns of consonant phonetic errors by Khmer preschool children can be simplified as follows: substitution errors > deletion errors > addition errors & distortion errors (error type-based) and velar consonant errors > alveolar consonant errors > palatal consonant errors (place of articulation-based).

### Consonant Phonological Errors Across the Three Age Groups

#### General Performance in Consonant Phonological Errors

Table 3 shows the means and standard deviations of phonological errors among Khmer-speaking children, highlighting significant differences across the three age groups. Specifically, children aged 3;0–3;11 exhibited the highest number of both regular (*M* = 136.07, *SD* = 221.95) and irregular (*M* = 107.46, *SD* = 96.93) phonological errors, with each type accounting for 53%–46% of the total errors (see Figure 3). This was followed, in turn, by those aged 4;0–4;11 (regular: *M* = 101.35, *SD* = 148.53; irregular: *M* = 89.69, *SD* = 75.84) and those aged 5;0–5;11, who showed the fewest errors (regular: *M* = 45.71, *SD* = 78.81; irregular: *M* = 62.23, *SD* = 86.96).

**Table 3.** Phonological consonant errors in the Khmer preschool children.

Error types	3;0–3;11 (*M* ± *SD*, *n* = 20)	4;0–4;11 (*M* ± *SD*, *n* = 20)	5;0–5;11 (*M* ± *SD*, *n* = 20)	*F* (*df*)	*p* value
Regular	136.07 ± 221.95	101.35 ± 148.53	45.71 ± 78.81	2, 39 = 1.12	.33
Irregular	107.46 ± 96.93	89.69 ± 75.84	62.23 ± 86.90	2, 36 = 0.89	.41

*Note.* *F* = one-way analysis of variance; *p* value = significance level.

**Figure 3.**
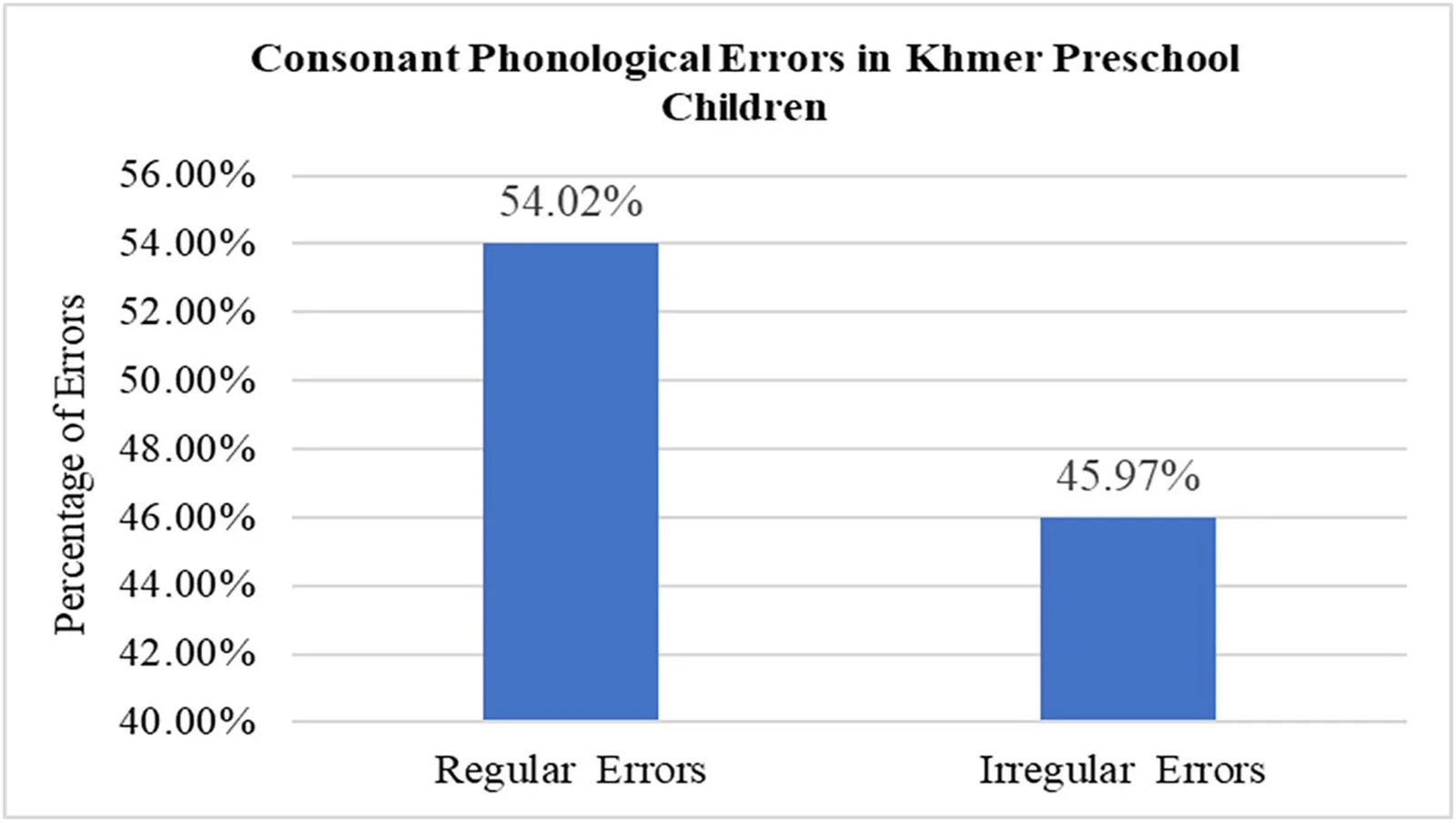
Percentage of regular and irregular errors of phonological error types in Khmer-speaking children during preschool contexts.

A one-way ANOVA was conducted to compare regular and irregular consonant errors across the three age groups, following the approach adopted in the General Performance in Consonant Phonetic Errors section. The results revealed no significant differences in age group for both regular errors, *F*(2, 39) = 1.12, *p* = .33, η^2^ = .05, and irregular errors, *F*(2, 36) = 0.89, *p* = .41, η^2^ = .04. Although not statistically significant, the descriptive results indicated a developmental decline in phonological errors with age.

As for the overall distribution of phonological error types, regular errors (*M* = 283.37, *SD* = 441.14) were slightly more than irregular errors (*M* = 259.38, *SD* = 219.28), with each type accounting for 54.02% and 45.97% of the total phonological errors (see Figure 4). However, a one-way ANOVA did not reveal any significant age-group differences, *F*(1, 25) = 0.03, *p* = .86, η^2^ = .01.

**Figure 4.**
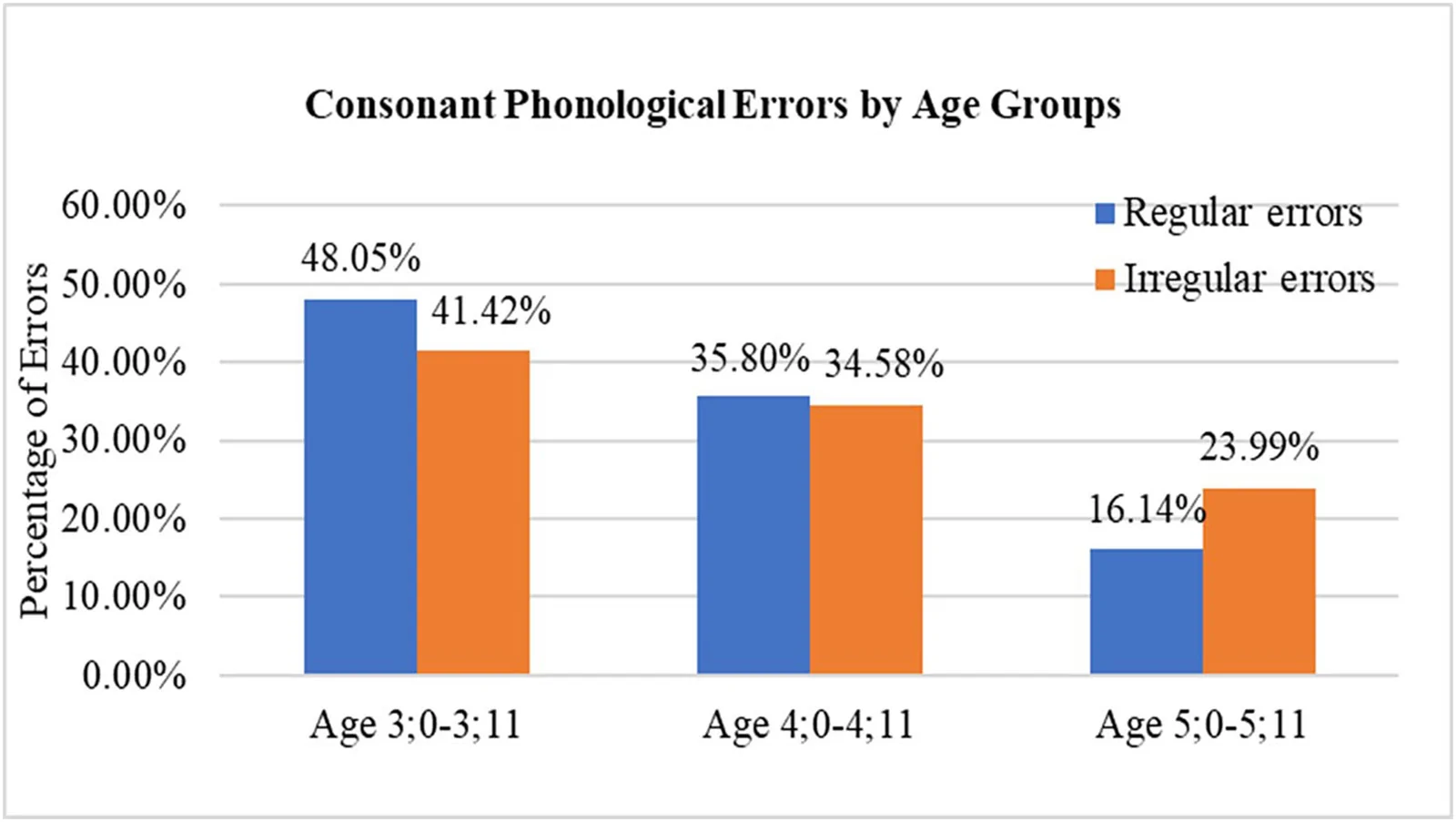
Percentage of phonological regular and irregular errors by Khmer-speaking preschool children across the three age groups.

Overall, younger children aged 3;0–3;11 had evidently more errors than older children, displaying the gradual development of Khmer consonant acquisition. Although not statistically significant, this trend suggests a steady reduction in both regular and irregular errors with age. This highlights the importance of early phonological awareness and interventions in kindergarten settings.

#### Patterns of Consonant Phonological Errors

Table 4 reports the percentages of regular and irregular phonological error patterns among Khmer-speaking preschool children aged 3;0–5;11. The results showed that Khmer preschool children produced regular errors (58.47%; *M* = 302.69, *SD* = 394.91) more frequently than irregular errors (41.53%; *M* = 215.00, *SD* = 147.70), revealing the basic pattern of consonant phonological errors by the children (in terms of phonetic regularity): regular consonant errors > irregular consonant errors (> meaning “more than”). However, ANOVA showed no significant difference in the error pattern (*p* = .49).

**Table 4.** Phonological patterns of consonant errors across age groups in Khmer-speaking preschool children.

Regular error patterns	3;0–3;11	4;0–4;11	5;0–5;11	Irregular error patterns	3;0–3;11	4;0–4;11	5;0–5;11
Final consonant deletion [kᵊbaːl] → [kᵊbaː]	0.89	6.02	3.10	Initial consonant deletion [ko:] → [o:]	12.50	1.15	0
Cluster reduction [sᵊʔaːt] → [ʔaːt]	38.81	34.53	45.27	Medial consonant deletion [kᵊdaɨkʰluɜn] → [kᵊdaɨkʰuɜn]	17.64	7.33	7.30
Weak syllable deletion [tɔkkaɛ] → [kaɛ]	9.95	11.33	13.95	Addition consonant [kɑŋhaː] → [tɑŋhaːl]	2.90	4.94	6.67
Fronting [k] → [t]	9.16	9.04	4.18	Medial consonant substitution [ʔɑŋkʊj] → [ʔɑŋtʊj]	9.08	11.28	6.04
Stopping [s] → [t]	7.14	8.98	16.12	Backing [t] → [k]	2.45	5.35	3.65
Palatalization [r] → [j]	2.79	2.29	1.39	Denasalization [n] → [t]	1.71	2.22	0
De-aspiration [pʰ] → [p]	2.61	2.29	2.17	Aspiration [k] → [kʰ]	20.47	11.44	14.23
Lateralization [r] → [l]	11.30	5.72	3.41	Nasalization [l] → [n]	1.48	4.94	3.27
Nasal substitution [n] → [m]	2.02	2.71	0.62	Simplification [bɑŋʔaɛm] → [pᵊʔaɛm]	8.36	21.49	41.30
Alveolarization [b] → [d]	0.89	1.38	0	Alveolarization substitution [d] → [t]	5.13	4.85	10.95
Velarization [c] → [k]	2.44	0	0	Final consonant substitution [daɜmtoʊn] → [daɜmtoʊn]	2.97	3.37	2.14
Aspiration substitution [kʰ] → [sʰ]	3.09	4.70	0	Glottalization [r] → [h]	4.61	16.96	4.03
Stopping of frication [d] → [s]	0.71	1.02	0	Labialization substitution [b] → [p]	2.45	4.61	0
Syllable substitution [dɑŋ] → [dɑn]	8.15	7.35	9.76				

*Note.* Values represent percentages of Khmer children within each group producing the specified regular and irregular error patterns. Examples are transcribed in International Phonetic Alphabet. Age groups are categorized as follows: 3;0–3;11, 4;0–4;11, and 5;0–5;11 (years;months).

Regular errors were more frequent than irregular errors across all age groups. Children aged 3;0–3;11 had the highest frequencies in both regular (*M* = 119.07, *SD* = 165.41) and irregular (*M* = 95.00, *SD* = 84.35) errors. However, the older age groups (4;0–5;11) displayed a significant reduction (regular: *M* = 92.39, *SD* = 117.39; irregular: *M* = 73.84, *SD* = 71.88).

Similar to the previous section, a repeated-measures ANOVA was conducted to compare the mean consonant phonological error patterns across the three age groups for the data stemming from a 2 (regulars and irregulars) × 3 (aged: 3;0–3;11, 4;0–4;11, and 5;0–5;11). The results indicated a significant main effect of error types, *F*(1.97, 25.72) = 6.90, *p* < .00, η^2^ = .34, with significant differences observed among the 3;0–5;11 age group (all *p*s < .02). Yet, no significant differences were found within the 3;0–4;11 age group (all *p*s = 1.00). In contrast, irregular errors showed no main effect, *F*(1.81, 21.76) = 0.36, *p* = .70, η^2^ = .02. Additionally, no age-group differences were found within aged 3;0–5;11 (all *p*s = 1.00).

Inside the regular errors, there occurred a pattern that the younger group (aged 3;0–3;11) produced the highest rate of final consonant deletion (0.89%), lateralization (11.30%), and initial consonant deletion (12.50%), consistent with early-stage phonological development in other languages (e.g., Dyson & Amayreh, 2000; Joye et al., 2022; Liu & Chien, 2020; Preston & Edwards, 2010). These errors gradually decreased with age, demonstrating a developmental trajectory toward more accurate speech production. In addition, some errors appeared to keep consonant across the age groups. For instance, cluster reduction showed little variation from age 3;0–4;1, but became the highest frequent error type in the three age groups (38.81%–45.27%). Stopping increased steadily with age, from 7.14% at age 3;0–3;11 to 16.12% at age 5;0–5;11. However, simplification showed the most pronounced age-related rise, from 8.36% in the age of 3;0–3;11 to 41.30% in the age of 5;0–5;11 (see Table 4 for the distribution of phonological error patterns across all age groups; also see Supplemental Material S2). These findings demonstrate that not all errors follow a linear path of resolution, and some errors that did not occur earlier in speech development emerged in the later stages of speech development.

By contrast, among the irregular errors, a different pattern emerged: Glottalization errors peaked at 16.96% in the 4;0–4;11 age group, while alveolarization error substitutions increased to 10.95% in the oldest age group (5;0–5;11). These two errors were the most frequent irregular error types, in contrast to the expected developmental trajectory. These findings suggest that these errors may point to language-specific articulatory difficulties or reflect atypical acquisition pathways in Khmer phonology. In brief, the patterns of consonant phonological errors by Khmer preschool children were like this: regular consonant errors (i.e., initial consonant deletion > lateralization > final consonant deletion by children aged 3;0–3;11) > irregular consonant errors (i.e., glottalization for children aged 4;0–4;11 and alveolarization substitution for children aged 5;0–5;11).

## Discussion

The present study adopted the picture-naming task (Dyson & Amayreh, 2000) to systematically investigate the consonant articulating errors in Khmer-speaking preschool children. The results revealed that Khmer-speaking children exhibited age-related variations in consonant error types and error patterns, showing general agreement with patterns observed in other languages. Specifically, phonetic error occurred more frequently than phonological errors across all three age groups, with more errors among the youngest age group. The following discusses the potential factors motivating the consonant articulating errors and the specific error patterns observed in Khmer-speaking preschool children.

### Positive Correlation Between Age and Consonant Articulating Accuracy

One key finding of this study is a significant difference in consonant articulating accuracy across the age groups of Khmer preschool children. The percentages of correct consonants score illustrated a clear progressive increase with age, with older children (71.68%; ages 4;0–5;11) consistently demonstrating higher phonological accuracy than younger children (29. 30%; ages 3;0–3;11) among the three age groups (see Supplemental Material S2 for more detailed information). This suggests that age plays a substantial role in shaping consonant accuracy in Khmer children's speech sounds.

These findings are aligned with those of previous studies on speech sound errors across languages (e.g., Liu & Chien, 2020; Nathan et al., 2004; Nojavan-Pirehyousefan et al., 2022; Phạm & McLeod, 2019; Preston & Edwards, 2010). For instance, Nojavan-Pirehyousefan et al. (2022) reported that Azeri Turkish–speaking preschool children aged 3;0–3;11 made more consonant errors than those aged 4;0–4;11, revealing a clear age-related increase in consonant articulating accuracy. Although the improvement may vary across languages and study designs, the patterns of speech sound development appear to follow a sigmoid rather than a strictly linear curve (Macrae & Tyler, 2014).

In addition, the data from naming tasks showed a significant positive correlation between age and consonant accuracy, suggesting that younger children 3–4 years of age are more severely impaired in speech sound production than those 5 years of age (see Supplemental Material S2). This trend highlights the importance and urgency of close monitoring and support from parents and caretakers of younger children during speech development.

With regard to clinical implications, ASHA (2014) and Goldman and Fristoe (1969) suggest that the percentage of correct consonants between 85% and 100% is normal. At the same time, less than 50% is more serious and requires clinical attention. In the data we investigated, four children scored below the 5% threshold, and some others fell below the 50% threshold (see Figure 5). Obviously, it is urgent to have these children go to either a hospital or a speech-language pathology center to receive special linguistic intervention and speech therapy.

**Figure 5.**
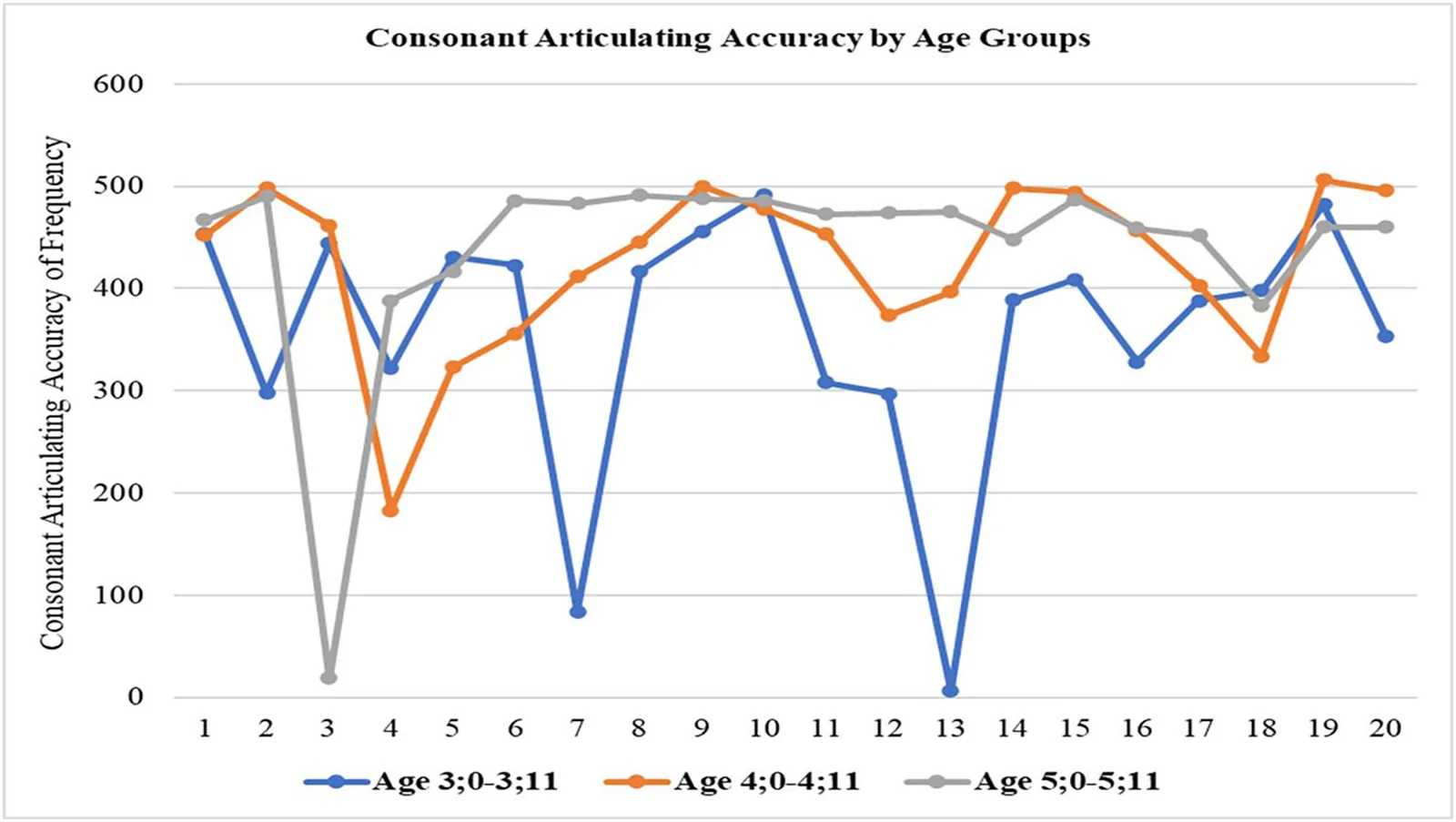
The percentages of consonant articulating accuracy in Khmer children by age groups (3;0–3;11, 4;0–4;11, and 5;0–5;11 [years;months]). Accuracy was calculated as the percentages of correctly produced consonants divided by the total number of attempted consonants. Each error bar points out to standard deviation.

### Phonetic Errors by the Khmer Preschool Children

#### The Panorama of Phonetic Errors Across the Three Age Groups

Children with consonant articulating errors have difficulties in various aspects of speech sound production (Preston & Edwards, 2010). As shown in the present study, Khmer-speaking preschool children aged 3;0–5;11 exhibited two primary types of phonetic errors: substitution and deletion. The error types were consistent with the findings in other languages (Dyson & Amayreh, 2000; Liu & Chien, 2020; Macrae & Tyler, 2014; Phạm & McLeod, 2019).

According to our test, the frequency of phonetic errors varies with age. Children aged 3;0–3;11 showed significantly more substitution errors than those aged 4;0–4;11 and 5;0–5;11. In addition, children aged 4;0–4;11 had notably more substitutions than those aged 5;0–5;11. These findings are consistent with Phạm and McLeod (2019), who observed a similar pattern among Vietnamese-speaking children, and with Liu and Chien (2020), who found substitution to be the most frequent error type among Mandarin-speaking preschool children.

In terms of deletion errors, children aged 3;0–3;11 produced more deletions than older children, with 5-year-olds exhibiting the least deletions, suggesting a developmental reduction in this error type with age. This result is similar to that of Macrae and Tyler (2014), who reported higher deletion rates in English-speaking preschool children.

Furthermore, our study showed an overall trend that substitution errors occurred more frequently than deletions among all age groups. These findings are parallel to those in other languages, including English (Macrae & Tyler, 2014) and Portuguese (Yavas & Lamprecht, 1988). Addition and distortion were rare (< 1%), aligning with Liu and Chien's (2020) view that such errors are less prominent in kindergarten children.

#### Patterns of Phonetic Errors Across the Three Age Groups

Children with speech sound errors often experience difficulties in producing consonants in their language, resulting in the so-called consonant phonetic errors. Although many errors were resolved some years ago, the affected children failed to achieve the correct speech sound patterns in their later stages of language acquisition (Preston et al., 2013). These persistent errors are closely associated with articulatory issues and may affect both speech development and early literacy skills (Eernisse, 2021; Ueda et al., 2015).

Our picture-naming tasks showed that Khmer-speaking preschool children made more phonetic errors. Among these, substitution errors occurred more than deletion errors among Khmer-speaking preschool children age 3;0–5;11. These errors mainly involved substituting alveolar [t], [s], and [r], palatal [c], and velar [k] consonants (see Table 2) and deleting alveolar consonants [r], [t], and [n] (see Table 2), in line with the findings in McLeod and Crowe (2018). The persistence of alveolar consonant [r] error within age groups was particularly notable among Khmer-speaking preschool children, consistent with the studies by Stoel-Gammon (2011), Yavas and Lamprecht (1988), and Yavaş and Kehoe (2024). This discovery, for one thing, highlights the articulatory difficulty of [r] in Khmer phonetics and supports the cross-linguistic evidence that [r] is typically one of the latest sounds to be acquired for the other (Kopečková et al., 2023; McLeod & Crowe, 2018; Yavas & Lamprecht, 1988).

In our test, the phonetic errors systematically showed a clear age-related trend among Khmer-speaking preschool children aged 3;0–5;11. Substitution error patterns occurred more frequently than deletion error patterns across the three age groups. Specifically, these errors were the most prevalent in the youngest age group (3;0–3;11), with their frequency decreasing progressively in older groups aged 4;0–5;11, suggesting an age-related decline in articulatory difficulties as children's speech develops (see Table 2). The above findings are consistent with children's speech developmental patterns observed in other languages, including English (Brosseau-Lapré & Roepke, 2019), Arabic (Dyson & Amayreh, 2000), German (Fox & Dodd, 2001), Vietnamese (Phạm & McLeod, 2019), and Portuguese (Yavas & Lamprecht, 1988). According to the previous studies, producing fricatives and liquids requires refined articulatory control (Phạm & McLeod, 2019; Yavas & Lamprecht, 1988) and is prone to both substitution and deletion in early speech production (Dyson & Amayreh, 2000; Fox & Dodd, 2001). The persistence of errors on [r] in Khmer is consistent with cross-linguistic findings indicating that these sounds require complex lingual gestures and are mastered later than stops and nasals (Preston et al., 2013; Ueda et al., 2015). Conversely, stops and nasals ([b], [p], and [m]) were more stable in speech production. These phenomena support the claim that sounds involving less precise gestures are acquired earlier (Cheek et al., 2001; Eernisse, 2021).

The participants' substitution of velar [k] with bilabial [p], alveolar [t], or glottal [ʔ] consonants indicated a simplification strategy. This result is consistent with observations in other languages, where glottalization is a default replacement in immature speech (Studdert-Kennedy, 2014). Such a substitution may reflect both the articulatory constraints and the structural properties of Khmer phonology.

To summarize, the substitution and deletion errors demonstrated that Khmer-speaking preschool children's phonetic errors followed general cross-linguistic patterns shaped by both universal articulatory constraints and language-specific phonological structures. However, the factors such as dialectal variation, socioeconomic background, and discrepancies in linguistic input may contribute to individual differences in consonant articulation. Therefore, these results should be interpreted with caution and in association with the context the consonant errors appear in.

### Phonological Errors in the Khmer Preschool Children

#### The Panorama of Phonological Errors Across the Three Age Groups

Phonological errors tend to occur gradually, and preschool children may incorrectly select speech parameters, resulting in both regular and irregular phonological patterns (Dodd et al., 1997). In the current study, Khmer preschool children aged 3;0–5;11 exhibited a wide range of phonological errors (see Figures 6 and 7), consistent with previous findings from cross-linguistic studies (Liu & Chien, 2020; Phạm & McLeod, 2019). These error trajectories reflect the developmental variability in phonological processing and articulatory control. Although no significant difference was found among the three age groups regarding the frequency of error types, age-related discrepancy is obvious, providing implications for both speech sound acquisition and clinical phonetic therapy (Preston & Edwards, 2010).

**Figure 6.**
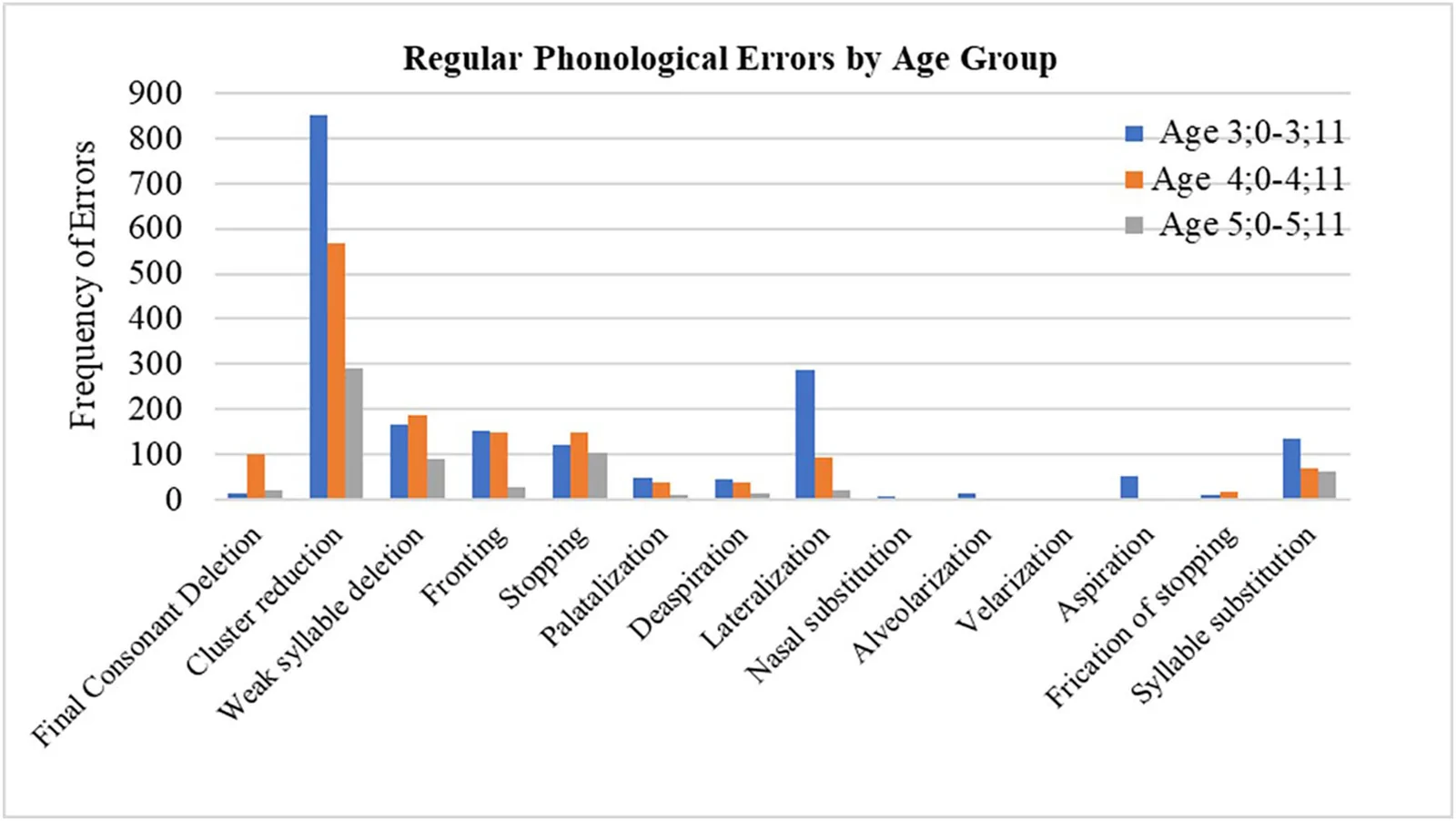
Frequency distribution of regular phonological errors in Khmer-speaking children across the three age groups (3;0–3;11, 4;0–4;11, and 5;0–5;11 [years;months]). Each bar represents the total number of observed error instances per error types. Age-related difference demonstrates developmental trends in error patterns.

**Figure 7.**
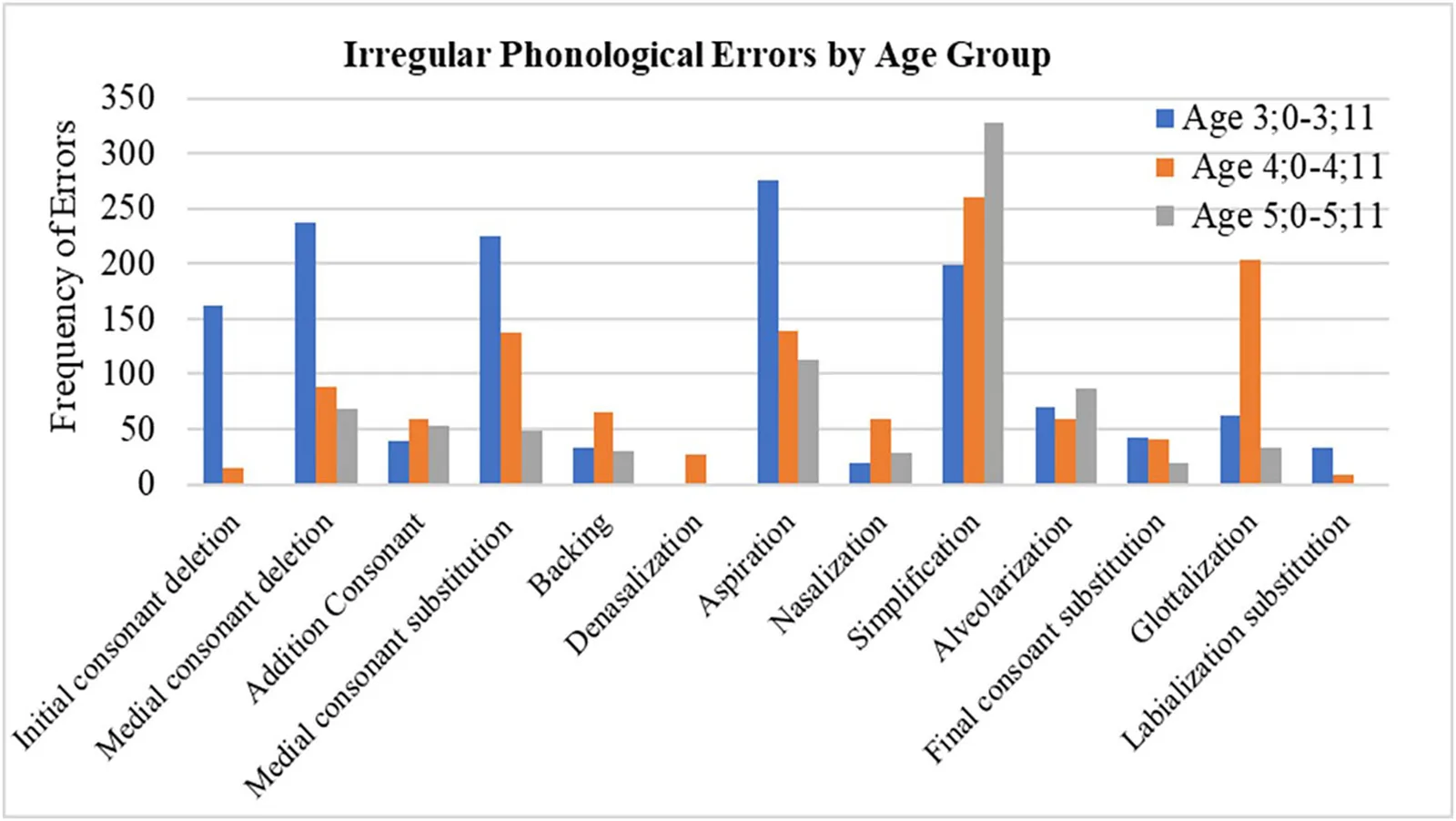
Frequency distribution of irregular phonological errors in Khmer-speaking children by the three age groups (3;0–3;11, 4;0–4;11, and 5;0–5;11 [years;months]). Each bar represents the total number of observed errors instances per error types. Age-related differences demonstrative developmental trends in error patterns.

Regular phonological errors reflect the normal development of consonant articulation that emerge predictably in children's speech. The observed errors in this study were consistent with the findings reported in previous studies using picture-naming tasks to assess speech sound errors in preschool-aged children (e.g., Brosseau-Lapré & Roepke, 2019; Fox & Dodd, 2001; Liu & Chien, 2020; Phạm & McLeod, 2019; Preston & Edwards, 2010). The results revealed that younger children exhibited a higher frequency of regular errors, which gradually decreased with age, indicating a typical pattern of developmental progression.

Among these phonological errors, weak syllable deletions were the most frequent. For example, the targeted word [srɒkaːniɜʔ] was frequently produced as [kaːniɜʔ] (dragon fruit). Other commonly observed errors included nasal substitution, cluster reduction, and final consonant deletion, particularly prevalent among younger children aged 3;0–3;11, which is similar to the error pattern documented in Vietnamese-speaking children and Mandarin-speaking children (Liu & Chien, 2020; Phạm & McLeod, 2019). Example [ŋuːttɘt] (take a shower) was realized as [muːttɘt], [pʰtɛah] (home or house) as [tɛah], and [kɑnteːl] (mat) as [kɑnteː]. These illustrative cases indicate age-related simplification trends, aligning with universal phonological acquisition patterns, in which children simplify complex structures early on (Dodd, 2013). In addition, fronting and stopping were observed across the three age groups, but were more prominent in younger children. For example, [koʊn] was used for [toʊ] (kid) and [srɛj] for [trɛj] (girl). These errors have been widely reported in children's speech in other languages (e.g., Brosseau-Lapré & Roepke, 2019).

Fewer frequent errors, including palatalization, lateralization, velarization, syllable substitution, and stopping frication, were evident in this study. As an example, [bɑŋkɒːŋ] was replaced by [tamtɒːŋ] (shrimp) and [sᵊdap] was produced as [sdap] (listen; see Table 4). Although rare, these error types are clinically relevant (Bowen, 2023; Hodson & Paden, 1981), as they can signal emerging atypically speech difficulties in Khmer-speaking children during early childhood. These results are consistent with broader developmental research on speech sound errors (Bowen, 2023; Dodd, 2013; Dunn & Davis, 1983; Hodson & Paden, 1981; Smith & Robb, 2005), intensifying the point that regular phonological processes are typical in early childhood but gradually resolve with age. The current data underscore the importance of early screening and tailored phonological intervention during kindergarten to support the development of nominative speech.

Unlike regular phonological errors, irregular phonological errors suggest atypical speech development in children's speech. In our survey, younger children aged 3;0–3;11 made more irregular errors as compared to the older peers. Although not statistically significant, the trajectory reflected results similar to the theoretical assumption that these errors may indicate underlying phonological processing deficits (Preston & Edwards, 2010), disrupting phonological representation rather than articulatory deficits alone (Preston et al., 2013).

The most frequently observed irregular phonological process was aspiration, as in [saɜc] realized as [sʰaɜt] (meat). Other frequently occurring errors were alveolarization, final consonant substitution, and simplification (see Table 4), particularly in older children. These findings are similar to those reported for English-speaking and Mandarin-speaking children (Dodd, 2013; Liu & Chien, 2020). Additionally, younger children more often exhibited initial consonant deletion and medial consonant deletion, as in [koː] for [oː] (cow) and [kᵊdaɨkʰluɜn] for [daɨkʰuɜn] (have a fever). As a rule, some irregular errors may decrease or disappear with age, while others persist, highlighting deeper phonological processing issues (Smith & Robb, 2005).

Additionally, consonant addition was observed across the three age groups but occurred less frequently than aspiration or deletion. Rare errors such as glottalization, backing, and denasalization (e.g., [riɜn] for [hiɜn] or [liɜn] (learn) and [rʊt] instead of [kʊt] (ran)) appeared sporadically. Labialization (see Table 4) occurred occasionally among 5-year-olds but was not widespread. These less common errors align with the findings in Dunn and Davis (1983) and Hodson and Paden (1981), which stress the need to identify atypical phonological patterns early in development.

While many irregular phonological errors show signs of resolution/absence with age, their persistence in some children may signal more complex difficulties in phonological processing. These results underscore the importance of developing Khmer-specific diagnostic tools and longitudinal research to support effective age-relevant intervention plans.

#### Patterns of Phonological Errors Across the Three Age Groups

Phonological error is the second type of consonant errors observed in the children's speech production. These errors typically signify inherent deficiencies in children's ability to organize and process the phonological structure of their language (Dodd, 2013; Hulme & Snowling, 1992). Children with phonological errors often display significant deficits in awareness of speech sounds, which, in turn, can affect broader language development skills (Bowen, 2023; Preston et al., 2013).

In our test, regular errors occurred more frequently than irregular phonological errors among Khmer-speaking preschool children aged 3;0–5;11. Regular phonological errors are consistent, rule-governed patterns that shape particular classes of sounds or sound sequences in speech (Reed, 1989). These patterns are considered developmentally typical when they are produced by at least 5% of the children at any given age (Graitcer & Gentry, 1981). For example, our children frequently substituted the aspirated velar [kʰ] with a fricative [sʰ], a regular pattern potentially attributed to simplification of the articulation process. The consistent presence of this pattern across age groups indicates that it is part of the normative phonological development in Khmer.

In contrast, irregular phonological errors were less consistent and did not conform to phonological rules as expected. Such inconsistent patterns are rarely observed in typically developing children and are often idiosyncratic (Graitcer & Gentry, 1981; Preston & Edwards, 2010). For instance, initial consonant deletion ([ko:] realized as [o:]) and backing substitution ([t] replaced by [k]) were identified in some Khmer preschool children, although they were inconsistent among speakers. These error patterns align with the well-documented trend of phonological errors across languages (Dodd & Bradford, 2000) and also with clinical red flags for phonological impairments in children's speech sounds during the preschool years (Dodd et al., 2024; Grunwell, 1988).

The age-related trend in our data set offers valuable insights into phonological development. Regular error patterns were more frequent than irregular error patterns across the three age groups within Khmer-speaking preschool children aged 3;0–5;11. Notably, simplification and cluster reduction increased substantially with age, rising from 23.59% in the 3;0–3;11 age group to 43.29% in the 5;0–5;11 age group. This divergence from typical developmental expectations may indicate persistent phonological difficulties in older children (see Table 4). Velar consonants, particularly [k] and [kʰ], were disproportionately affected by both regular and irregular processes, including fronting, aspiration, and deletion. These results are in line with cross-linguistic studies on the acquisition of velars owing to their greater articulatory complexity (Ingram & Babatsouli, 2024; Yavaş & Kehoe, 2024).

Velar substitution with alveolar or glottal consonants occurred inconsistently, reflecting a cross-linguistic tendency toward simplification of the articulatory process. However, the persistence of irregular substitutions, including glottalization and atypical medial consonant changes, may result in deeper phonological processing. These patterns underscore the clinical need to differentiate between phonological delay and phonological disorders, as the implications for treatment vary considerably.

These results have significant implications for speech-language assessment and intervention. The co-occurrence of regular and irregular processes indicates that a differentiated intervention approach is required (Wolk et al., 2016). While regular phonological errors may be remediated through naturalistic and developmental approaches, irregular phonological errors often require targeted and intensive therapy (Aiello et al., 2025). Given the high frequency and complexity of velar-related errors, special attention should be paid to targeting velar production in therapeutic interventions. This focus may improve overall speech intelligibility and contribute to greater phonological stability in preschool children.

From a linguistic perspective, the present study provides further evidence of language-universal and language-specific aspects of phonological development. The study demonstrates the importance of culturally and linguistically appropriate diagnostic tools to support the early identification and treatment of speech sound errors among underrepresented language groups, such as Khmer speakers.

### Pedagogies and Clinical Implications for Language Acquisition

This study is the first pioneering investigation of consonant articulating errors by Khmer-speaking preschool children. By identifying specific error types and patterns across different age groups, our test was designed to provide a unique insight for understanding Khmer speech sound acquisition and for designing effective pedagogical and clinical interventions for Khmer-speaking preschool children.

First, children aged 3;0–3;11 exhibited a higher frequency of both phonological and phonetic errors than those aged 4;0–5;11. This developmental pattern highlights the importance of early treatment of speech sound errors or even disorders. As Inguaggiato et al. (2017) stressed, early treatment can exploit the plasticity of the developing phonological system, underscoring the need for timely support in early education (Ceron et al., 2013; Gierut, 1998). Educators should collaborate closely with speech-language therapists to generate phonological treatment strategies tailored to specific error patterns in early kindergarten curricula. For older children, the pedagogical focus should shift to refining and stabilizing phonological systems and to placing a greater emphasis on the phonetic hierarchy in children's speech production. In particular, phonetic errors may respond well to evidence-based techniques such as minimal pair therapy, auditory discrimination activities, and self-monitoring practices (Asaro-Saddler, 2016; Shields & Hopf, 2024).

Second, the findings suggest a critical role for early and accurate diagnosis of speech sound errors in Khmer-speaking preschool children. Speech-language therapists played a pivotal role in identifying these errors and developing age-appropriate, linguistically informed intervention plans. According to Buac and Jarzynski (2022) and Harris et al. (2014), assessment tools must be both culturally and linguistically responsive to ensure diagnostic accuracy in diverse populations. A good understanding of consonant error typologies in the Khmer language can inform the development of culturally appropriate assessment instruments, thereby facilitating the distinction between typical developmental errors and clinically significant speech errors. Early clinical intervention is not only beneficial but also important for preventing long-term social communication and literacy issues.

Third, the current study calls for the urgent need in the specialized training for speech-language therapists who work with Khmer-speaking preschool children. Such training should provide a comprehensive understanding of Khmer phonology and the common developmental error patterns. As Ambreen and To (2022) and Zydorowicz et al. (2025) indicated, multilingual and bilingual clinicians must be trained to recognize cross-linguistic influences on children's speech sound production. Training programs should also prioritize multilingual competence to address the needs of linguistically diverse children in Cambodian communities. Adaptationally, regular screening should be conducted in preschools to identify children with speech sound errors. In doing so, therapists can better support these children, ensuring their successful communication in the community and literature skills.

## Conclusions

This study adopted a picture-naming task to describe the consonant errors articulated by Khmer-speaking preschool children among three age groups from 3;0 to 5;11. The results show that phonetic errors occur more frequently than phonological errors across the age groups. Specifically, the children aged 3;0–3;11 displayed a higher frequency of both phonetic and phonological errors than those aged 4;0–5;11, despite no significant difference in statistics. This result is consistent with the findings in other high-source languages including English. Within the two types of consonant errors, Khmer preschool children basically observe the patterns as below:

Phonetically, substitution errors > deletion errors > addition errors & distortion errors (error type-based) and velar consonant errors > alveolar consonant errors > palatal consonant errors (place of articulation-based);

Phonologically, regular consonant errors (i.e., initial consonant deletion > lateralization > final consonant deletion by children aged 3;0–3;11) > irregular consonant errors (i.e., glottalization for children aged 4;0–4;11 and alveolarization substitution for children aged 5;0–5;11).

These patterns suggest that children had both articulatory difficulties and phonological processing deficits, with younger children tending to produce the majority of speech sound errors. This was followed in turn by children whose errors persisted with age, children who showed partial resolution, and age-matched typically developing children. Overall, the study's findings highlight the risk of language delays or disorders among Khmer-speaking preschool children.

This study has theoretical and practical implications. Theoretically, it provides a preliminary description of phonological errors in Khmer, a typical low-source language, so that the audience can gain a fundamental understanding of Khmer consonant articulation. Practically, the results can inform clinicians about specific error types to prioritize diagnoses and intervention plans for early assessment and intervention. Educators should identify children at higher risk of speech errors and work closely with therapists to ensure they receive timely support in speech learning. Additionally, it is suggested that children with severe speech sound errors receive early phonetic training to pronounce their mother tongue correctly, which should be taken as a general rule for all phonetically abnormal children, particularly those in low-source language communities.

However, this study also has several limitations. First, the small sample size may affect the generalizability of our findings. Future studies should enlarge the sample size by recruiting more diverse participants to ensure that results are more representative statistically. Second, the lack of significant differences in error scores across age groups poses issues in capturing developmental variation, an issue to be considered in future task design. Third, although the picture-naming tasks triggered the production of all consonants, the phonetic contexts have not yet covered all situations in Khmer.

Further research should examine the developmental trends of children with speech sound errors, including early longitudinal interventions. A detailed analysis of the age-specific error patterns and their persistence over time is required.

## Data Availability Statement

The data sets supporting the results of the present study are available in Supplemental Materials S1 and S2. Supplemental Material S3 presents the raw speech-sound productions collected from Khmer-speaking preschool children aged 3;0-5;11, as well as the targeted words and corresponding child-specific IPA transcriptions used in the analysis, respectively.

## Supplementary Material

10.1044/2026_JSLHR-25-00756SMS1 Supplemental Material S1 Data set and coding scheme for speech sound productions in Khmer-speaking preschool children.

10.1044/2026_JSLHR-25-00756SMS2 Supplemental Material S2 Data set and coding system for speech sounds by Khmer-speaking preschool children.

10.1044/2026_JSLHR-25-00756SMS3 Supplemental Material S3 Raw speech-sound productions collected from Khmer-speaking preschool children aged 3;0-5;11, as well as the targeted words and corresponding child-specific IPA transcriptions used in the analysis, respectively.

## Footnote

1 In addition to the first author, other three testers were the kindergarten school teachers in Siem Reap and Kampong Cham province, Cambodia.

2 Although the average number of phonological errors was higher (*M* = 90.57) than that of phonetic errors (*M* = 82.99) for each child, the total number of phonetic errors was greater (*n* = 9,959 vs. *n* = 7,336), due to the large sample size. Accordingly, phonetic errors comprised a greater proportion of the total errors (57.58%) compared to phonological errors (42.42%).

## Appendix A

### Consonants of Khmer in Terms of Place and Manner of Articulation

**Table:**

Manner place	Bilabial	Alveolar	Palatal	Velar	Glottal
Stop	p b	t d	c	k	ʔ
Nasal	m	n	ɲ	ŋ	
Trill		r			
Fricative		s			h
Approximant	v		j		
Lateral approximant		l			

## Appendix B

### Words Adapted by Khmer-Speaking Preschool Children in the Experiment

**Table:**

Khmer word	Meaning	Khmer word	Meaning	Khmer word	Meaning
maʔ	Mother	crʊɲ	Chopping board	kɒmpɨh	Crayfish/small shrimp
koʊn	Babay	slɜk	Leaf	liɜŋcaːn	To wash the dishes
baː	Father	tʰpoal	Cheek	dɒmrɛj	Elephant
ɲam	Eat	smaː	Shoulder	srɒmaɔc	Ant
hɑt	Tire	rʊt	Ran	koʊnkʰmeːŋ	Young children
pʰtɛah	House	kᵊdaː	Board	tɘɨriɜn	To go to study
kʰʊm	Bee	kʰcɑŋ	Snail	TɘKsɔt	Pure water
tʰnam	Medicine	sɛh	Horse	kɑnteːl	Mat
trɛj	Fish	tɔk	Table	TɘKmᵊteh	Chili sauce
caːp	Bird	nʊm	Cake	turᵊsap	Mobile phone
srɛj	Girl	mᵊrec	Pepper	bɒntoat	Ruller
prɛeh	God	sraː	Wine	kɑnsaɛŋ	Scarf
pʰlɔm	Bloom	spɛj	Cabbage	sabuː	Soap
prɔh	Boy	lᵊhɔŋ	Papaya	ʔaʔsɒː	Script
muɜk	Hat	kʰtɜm	Garlic	cʊndaɜ	Step
cʰkaɛ	Dog	muɜj	Number 1	tɘkdɑhkoː	Milk
jiːɜj	Grandmother	piː	Number 2	meːʔambaɨ	Butterfly
taː	Grandfather	bɜi	Number 3	srɒkaːniɜʔ	Dragon fruit
vɛj	Fight	buɜn	Number 4	turᵊtuɜh	TV
pʰsaː	Market	pram	Number 5	tʊmpɛaŋbaːjcuː	Grape
laːn	Car	prammuɜj	Number 6	kʰsaɛkrɒvat	Belt
riɜn	Study	prampiː	Number 7	tɔkkaɛ	Gecko
skɒː	Sugar	prambɜi	Number 8	ciŋcɑʔ	Lizard
pʰʔaɛm	Sweet taste	prambuɜn	Number 9	kɒnlaːt	Cockroach
tɪɲ	Buy	dɒːp	Number 19	krɒhɒːm	Red
tʰmɪɲ	Teeth	kʰlaŋ	Strong	ʔɒ:kʊn	Thank
kʰlaːc	Scare	sac	Meat	siʊphɘɨ	Book
kʰmaɔc	Ghost	kʰluɜn	Body	tɘkkᵊdaɨ	Hot water
kᵊʔɑːʔ	Cough	sŋaɔ	Soup	tɘktrɒcɛaʔ	Cold water
lᵊʔɒː	Good	kʰcaɨ	Shellfish	sɜtsɒʔ	Combing
pʰsɜt	Mushroom	svaːj	Mango	trɒbaɛc	Guava
cʰuːk	Lotus	baːj	Rice	ʔɜɨlɜk	Watermelon
dai	Hand	pʰɜk	Drink	bɛhdoʊŋ	Heart
kʰmaɨ	Black	dɔt	Burn	tɘkkɒmsieu	Kettle
sɒː	White	kʰnaɜj	Pillow	crɒmɔh	Nose
kʰieʊ	Blue	kruː	Teacher	trɒciɜʔ	Ear
lɨɜŋ	Yello	lʊj	Money	polih	Police
kɒː	Neck	pʰcuːɜ	Plow	moʊtoʊ	Moto
kɑŋ	Bicycle	srɜɨ	Rice	juənhɑh	Airplane
pʰkaː	Flower	kᵊdaːm	Grab	kataːp	Bag
sᵊʔaːt	Pretty	cʰmaː	Cat	kɑŋhaː	Fan
kᵊbaːl	Had	criɜŋ	Sing a song	niʔjiɜj	Speak
pʰlɘɨ	Thigh	sᵊdap	Listen	salaː	School
koː	Bovine	kʰuːɜ	Brain	dɑŋkɜɨ	Beetle grub
cruːk	Pig	sɒʔ	Hair	ʔɑŋkʊj	Sit
kʰlaː	Tiger	bɒŋkiːɜ	Shrimp	cʊmpuː	Wax apple
mᵊteh	Chili	bɒnlaː	Thorn	trɒkuɜn	water convolvulus
kʰaɔ	Pants	bɑŋʔaɛm	Dessert	ʔɑŋrɘŋ	Hammock
Cʰɨː	Sick	karaɛm	Ice cream	kᵊdaɨkʰluɜn	Fever
pʰtɜl	Cup/bowl	saɨmaːɨ	Rambutan	pʰkaː cʰuːk	Lotus flower
tʰliːɜʔ	Fall down	krupɛːt	Doctor	daɜc koːl	Nail
kʰmeːŋ	Baby	krɒdah	Paper	kahvɛe	Coffee
leːŋ	Play	kɑŋkaɛp	Frog	pɛaʔsᵊbaɛc cɘːŋ	Shoe
cᵊʔaɛt	Got full (eat)	rɔbɒːŋ	Fence	tʰurɛen	Durian
doʊŋ	Coconut	kambɜt	Knife	daɜmdoʊŋ	Coconut tree
pʰliɜŋ	Rain	tɘkkɒːʔ	Ice	daɜmtʰnaɔt	Palm tree
saɜc	Laugh	trɒsɑʔ	Cucumber	krɒbɛj	Buffalo
craɜn	Lots	karɔt	Carrot	kʰmaɨ dai	Pencil
pʰlɘːŋ	Light	sɑndaɛc	Bean	bɒnlaːɛ	Vegetable
kroʊc	Orange	nɔnoːŋ	luffa gourd	prɒcrʊj	Mole
duɜl	Fall down	tʊnsaːj	Rabbit	sɒseː	Write
cʰnaŋ	Pot	ʔɑndaːt	Tongue	kʰsaɛkɒː	Necklace
moan	Chicken	ŋuːttɘk	Take shower	slappriːɜ	Spoon
tʰviːɜ	Door	bɑŋkɒːŋ	Lobster	skɒːkroap	Candy
daɜ	Walk	ʔɑŋkrɒːŋ	Weaver ant	srɒlaɲ	Love

*Note.* IPA refers to the International Phonetic Alphabet. The table presents the stimulus words used in the present study, with their phonetic transcriptions and English glosses. These items were selected to present a range of speech sound production (phonetic and phonological) observed in Khmer-speaking preschool children.
